# Strategic replacement of soybean meal with local cotton seed meal on growth performance, body composition, and metabolic health status indicators in the major South Asian carp *Catla catla* for aquaculture

**DOI:** 10.1371/journal.pone.0296220

**Published:** 2023-12-22

**Authors:** Muhammad Haroon Aslam, Noor Khan, Mahroze Fatima, Muhammad Afzal Rashid, Simon J. Davies

**Affiliations:** 1 Department of Fisheries and Aquaculture, University of Veterinary and Animal Sciences, Lahore, Pakistan; 2 Department of Animal Nutrition, University of Veterinary and Animal Sciences, Lahore, Pakistan; 3 Aquaculture and Nutrition Research Unit (ANRU) Carna Research Station, School of Natural Sciences and Ryan Institute, University of Galway, Galway, Ireland; Tanta University Faculty of Agriculture, EGYPT

## Abstract

This study assessed the effect of substituting soybean meal (SBM) with cotton seed meal (CSM) on different biological traits in thaila (*Catla catla*). Fish (n = 225) with an average initial body weight of 41.53±0.68 g were shifted into hapas (3 (L) x 2 (W) x 1 (D) m) in triplicate (15 fish/replicate). Hapas were divided into five dietary groups: 0CSM, 25CSM, 50CSM, 75CSM, and 100CSM diet treatments were administered diets for a period of 90 days. SBM was replaced by CSM at the levels of 0, 25, 50, 75, and 100%. The results showed that fish survival and growth performance were not affected by the inclusion of CSM in the fish diet up to 50% as a replacement of SBM, but higher replacement levels showed a negative effect. Similarly, body composition and most of the muscle amino acid profiles were not affected significantly (*P>0*.*05*) by replacing SBM with CSM. Digestive enzyme activities were significantly (*P<0*.*05*) decreased by increasing the level of CSM in the fish diet. Alanine transaminase (ALT) and aspartate transaminase (AST) levels increased significantly (*P<0*.*05*) with increasing dietary CSM levels, while alkaline phosphatase (ALP) levels remained the same. Malondialdehyde (MDA) and catalase (CAT) activity decreased significantly (*P<0*.*05*), but superoxide dismutase (SOD) activity showed no change. For the intestine, the villus height to villus width ratio and thickness of Tunica muscularis were also better in 25CSM, and their values decreased as the CSM inclusion level increased in the fish diet. In conclusion, SBM could be replaced partially (up to 50%) with CSM without compromising growth performance, whole body proximate composition or immunity of *C*. *catla*.

## Introduction

The fisheries sector in Pakistan has a significant impact on food security by decreasing the reliance on terrestrial protein such as chicken, beef, and mutton, thus fulfilling an essential role. Currently, there is a dire need to do much work in this sector to flourish its expansion, as more profit could be generated by exporting seafood to other countries. In 2021, total fish production was 178.1 million metric tons, which increased to 184.6 million metric tons in 2022 [1]. In animal production systems, feeding cost contributes approximately 70% of the total cost of production. Therefore, profit in fish production systems could be increased by efficient feeding and management. Soybean meal is commonly acknowledged as a highly beneficial protein source derived from plants. Its renown is attributed to its high protein content, comprehensive amino acid profile, and favourable digestibility [2]. Pakistan relies on imports for SBM, as it is not self-sufficient in its production and utilization. Presently, the cost of SBM is witnessing a notable increase, and the recent impact of the COVID-19 pandemic has disrupted marketing and distribution networks. Consequently, farmers face challenges in acquiring essential supplies from abroad. Similar to other livestock sectors, researchers are actively exploring alternatives to SBM, aiming for cost-effective yet high-quality protein sources that do not compromise production performance in fisheries [3].

Cotton seed meal, the by-product of the cotton seed oil and cotton fibre industry, could be a suitable candidate in this regard, as Pakistan is producing cottonseed at higher levels than other oil seed crops. The use of cottonseed produced within Pakistan would be an ideal sustainable and viable alternative, and the total cottonseed production during 2022 was 2.12 million tonnes in Pakistan [4]. In addition, the cost of CSM is much less than that of SBM per unit protein, so it not only reduces the import pressure of SBM but also reduces the cost of production in our fisheries aquaculture economic strategy. For several years, CSM has been extensively utilized in formulating feed for various livestock animals [2]. The crude protein content of CSM can range from 23% to 53%, depending on the specific processing method employed to extract oil from the seeds [5]. The literature reveals numerous experiments that have been conducted to substitute fish meal and SBM with CSM in various cultured fish [2, 5–8]. In a 2-month feeding trial conducted by Liu et al. [9], the researchers investigated the impact of substituting dietary fish meal with CSM on the growth performance and selected health indicators of juvenile grass carp, *Ctenopharyngodon idellus*. Their findings provide support for the feasibility of incorporating up to 18% CSM before observing adverse effects. These effects include compromised growth, disruptions in digestive enzyme capacity, and a decrease in the gut integrity of carp. Recently, Stephen et al. [10] comprehensively discussed the importance of CSM and various processing technologies for utilization in aqua feeds. These authors considered many aspects both in terms of attributes such as the nutritional profile and the constraints associated with this commodity, including the variability of its antinutritional factors (ANFs). Although CSM is rich in protein, it does come with certain antinutritional factors, notably gossypol, a polyphenolic compound. Gossypol, being potentially toxic to fish and other animals, presents a hurdle to their consumption of CSM. The primary adverse impact of gossypol includes a loss of appetite and inhibited growth in animals [11], impairment in the reproductive system [12], and depression in immunity and haemoglobin levels [5, 13]. CSM also binds the essential amino acid lysine and reduces its bioavailability to animals [14, 15]. However, studies have shown that fish can endure higher levels of dietary free gossypol. Rainbow trout (250 mg per kg diet) [16], crucian carp (642 mg per kg) [7], and tilapia (180 mg per kg diet) [17] have demonstrated relatively higher tolerance for gossypol in their diets. This study aims to assess the influence of gradually substituting SBM with CSM to establish the finest replacement level of CSM in the diet of *C*. *catla* while ensuring that their growth performance and other biological parameters remain uncompromised. It is worth highlighting that this study stands as the pioneer in exploring parameters such as intestinal enzyme activities, antioxidant status, and gut morphology as indicators of metabolic health in *C*. *catla*, a commercially significant carp species.

## Materials and methods

### Ethical approval, experimental design, and conditions

The present research was conducted at the University of Veterinary and Animal Sciences, Lahore, Pakistan. The protocols and procedures of this study were approved by the animal use and animal care committee of the University of Veterinary and Animal Sciences, Lahore, Pakistan (DR/369). In a 90-day experimental period, a total of 225 fish (*C*. *catla*) with an average body weight of 41.53±0.68 g were stocked in 15 hapas (3 (L) x 2 (W) x 1 (D) m 1582.80 gallon water capacity) in triplicate (15 fish/replicate). Five dietary groups were designated 0CSM, 25CSM, 50CSM, 75CSM, and 100CSM. The control group (0CSM) was administered without CSM, while the diets of the 25CSM, 50CSM, 75CSM, and 100CSM groups were formulated by inclusion of CSM at levels of 25%, 50%, 75%, and 100%, respectively (Table 1).

**Table 1:** Ingredients and chemical composition of experimental diets.

Ingredients	Treatments				
	0CSM	25CSM	50CSM	75CSM	100CSM
Maize grain	20.00	20.00	20.00	20.00	20.00
Fish meal	10.00	10.00	10.00	10.00	10.00
Soybean meal	25.00	18.75	12.50	6.25	0.00
Cottonseed meal	0.00	6.25	12.50	18.75	25.00
Corn gluten 60%	7.00	7.50	8.00	8.50	9.00
Rice Polish	16.00	16.00	16.00	16.00	16.00
Wheat flour	15.00	14.50	14.00	13.50	13.00
Fish Oil	5.00	5.00	5.00	5.00	5.00
Vitamin premix^1^	1.00	1.00	1.00	1.00	1.00
Mineral mixture^2^	1.00	1.00	1.00	1.00	1.00
Total	100	100	100	100	100
Crude protein%	25.11	25.10	25.06	25.04	25.12
EE%	9.43	9.60	9.78	9.95	10.12
Crude fibre%	3.17	3.95	4.73	5.51	6.29
Ash%	5.53	5.58	5.64	5.70	5.76
Metabolizable energy (kcal/kg)^3^	3131	3227	3324	3420	3517
Lysine%	1.36	1.30	1.23	1.16	1.10
Methionine%	0.51	0.50	0.50	0.50	0.50

*0CSM: control without any CSM, 25CSM, 50CSM, 75CSM and 100CSM containing 25, 50, 75 and 100% CSM as replacement of SBM, respectively.

^1^_Vitamin A 15 M.I. U, Vitamin D3 3M.I. U, Nicotinic acid 25000 mg, Vitamin B1 5000 mg, Vitamin E 6000 IU, Vitamin B2 6000 mg, Vitamin K3 4000 mg, Vitamin B6 4000 mg, Folic acid 750 mg, Vitamin B12 9000 mg, Vitamin C 15000 mg, Calcium Pentothenate 10000 mg_

^2^_KH2PO4 479 mg/g, MgSO4.7H2O 153 mg/g, CoCl.6H2O 0.0816 mg/g, NaCl 51 mg/g, AlCl3.6H2O 0.255 mg/g CuSO4.5H2O 210.67 mg/g, FeSo4. H2O 100.67 mg/g, MnSo4.5HO 116.67 mg/g, ZnSO4.7H2O 121.33 mg/g CaCO3 316 mg/g and cellulose 65 mg/g._

^3^_ME (kcal/kg) = (2.25 × Crude protein %) + (4.0 × Ether extract %) + (9.0 × Nitrogen-free extract %) + (4.0 × Crude fibre %)._

CSM was procured from Sind Feed and Allied Products, located at Plot No. 19, Sector No. 16, Korangi Industrial Area, Karachi, Pakistan 75600, in the Sindh region of Pakistan. The gossypol contents in the CSM were assessed in terms of total gossypol, with a minimum quantification limit established at 20 mg/kg. Gossypol extraction was conducted using 3-aminopropan-1-ol coupled with a blend of propan-2-ol and hexane for the assessment of free gossypol or di-methyl-formamide for the determination of total gossypol. Following this, gossypol was transformed into gossypol-dianiline using aniline, and the optical density of the resultant compound was gauged at 440 nm. The determined gossypol level was 591.6 mg/kg, employing a modified version of the method described by Tchatchueng and Porte [18]. The SBM was sourced from South America, specifically Argentina. The solvent extraction of SBM was carried out by Ghousia Agro Products on Bahawalpur Road in Multan, Pakistan. The experimental feed was prepared using a Qidong Extruder and subsequently dehydrated in a hot air oven before being stored in airtight plastic bags. The physicochemical parameters of the water, including a temperature range of 24.9–28.7°C, pH levels between 7.4–8.6, and dissolved oxygen levels of 5.8–7.3 mg/L, were maintained throughout the entire duration of the experiment. The fish were fed twice daily (09.00 AM to 04.00 PM), with the amount of feed provided equivalent to 3% of their body mass. The feed quantity was adjusted to accommodate changes in body mass during the 14-day weighing inventory.

### Growth performance and body indices

The initial body mass was noted at the beginning of the experiment. Subsequent measurements were taken fortnightly, and the final measurement was noted at the end of the trial according to Hopkins [19]. The daily feed amount provided to the fish was weighed, and the amount of leftover feed (and mortalities) was also recorded. Other growth parameters, including body weight gain, specific growth rate (SGR), feed conversion ratio (FCR), and survival rate (SR), were calculated using the following equations:

Bodyweightgain(g)=finalbodyweight(g)–initialbodyweight(g)

FCR=Totaldryfeedintake(g)/(Wetweightgain(g)

SGR=[Finalweightgain–initialweight)/daysofgrowthtrial]×100

Survivalrate(%)=100×(finalfishnumber/initialfishnumber)

Liver sample weights were recorded to calculate hepatosomatic indices (HSI), while visceral weights were recorded to calculate the viscerosomatic index (VSI) using the following formulas:

$$HSI=\frac{wet\;weight\;of\;sample\;(g)}{wet\;weight\;of\;fish\;(g)}\times 100$$

$$VSI=\frac{Viscera\;weight\;(g)}{wet\;weight\;of\;fish\;(g)}\times 100$$

### Sample collection

At the conclusion of the trial, the fish underwent a 24-hour fasting period and were subsequently anaesthetized using MS 222, following the methodology proposed by Zheng et al. [20]. From each replicate, three fish were selected, euthanized (cranial percussion), and dissected to collect samples for the assessment of biological indices (HSI and VSI), three fish for amino acid profiling from muscle, and three fish for digestive enzyme activities from intestine. Three fish specimens were extracted from each replicate, appropriately labelled, and subjected to proximate analysis. Additionally, another set of three fish per replicate was randomly chosen for blood sampling, which was performed to analyse serum biochemical parameters and antioxidant enzyme activities. Serum was separated from the blood samples through centrifugation.

### Body composition

The entire fish underwent drying and subsequent proximate analysis, encompassing the determination of dry matter (DM), ether extract (EE), crude protein (CP), and total ash, following the AOAC [21] protocols. For the determination of DM, the samples were subjected to drying at 105°C to a final constant weight. The CP percentage was determined using the method of Kjeldahl, with CP calculated as N × 6.25. The EE (Ether Extractives) percentage was calculated using a Soxhlet apparatus. The total ash percentage was derived by subjecting the samples to a muffle furnace at 660°C.

### Amino acid profile of muscle

Muscle samples were hydrolysed at 110°C for 22 hr in 6 N HCl solution in a sealed tube following the method of Huang et al. [22]. Following hydrolysis, an automatic amino acid analyser (Hitachi, 835–50, Japan) was used for determination of the amino acid profile.

### Digestive enzyme activity

After the feeding trial, three fish from each replicate were randomly sampled, and the intestine was dissected and homogenized in Tris HCl buffer (0.1 M, pH 7.4, 1:9 ratio) for 15 minutes at 4°C on a tissue analyser at 6,000 g. The lipid fraction was removed, and the supernatant was recovered and stored at -20°C for later enzyme activity determination. The activities of amylase, protease, and lipase were determined as described by [23]. Intestinal protease enzyme activity was determined by making a substrate solution of 1% azoalbumin in 50 mM Tris-HCL (pH 7.5). The enzyme extract (10 μL) was mixed with buffer (0.5 mL; pH 7.5), and then 0.5 mL substrate solution was added. The samples were incubated at 25°C for 10 min. The reaction was stopped by adding 0.5 mL trichloroacetic acid and centrifuged at 14,000 × g for 3–5 min. The absorbance of collected supernatant aliquots was read at 366 nm [24]. To analyse the amylase activity, 1 mL of supernatant was mixed with 2 mL of starch phosphate buffer and incubated at 37°C for 30 min. After that, 3 mL of DNS solution was added and again incubated until a brown colour appeared. The solution was diluted by adding 4 mL of water to make a 10 mL volume. The optical density of the supernatant was measured at 540 nm [25]. For lipase activity, 50 μL of the extract solution was mixed with 0.5 mL of olive oil, 10 mM CaCl2 and 0.5 mL of phosphate buffer. Samples were incubated at 30°C for 30 min, and the reaction was stopped by adding 20 mL of ethanol. The fatty acids were estimated by 50 mM KOH [26].

### Serum biochemistry

For the determination of different blood biochemical constraints, aspartate aminotransferase (AST), alanine aminotransferase (ALT), and alkaline phosphatase activity (ALP) were determined by an automated biochemical analyser (Micro Lab-300).

### Antioxidant activity

According to the Chance and Maehly [27] approach, catalase (CAT) activity was measured by its capacity to lower the concentration of -H2O2 at 20 nm. Photo reduction inhibition of nitro blue tetrazole (NBT) according to the technique of Giannopolitis and Ries [28] was used to test superoxide dismutase (SOD) activity, and malondialdehyde (MDA) levels were determined by the addition of thiobarbituric acid and HCl, boiled for 25 min, cooled and centrifuged. Then, the MDA value was determined photometrically at 530 nm by the technique of Gatta [29].

### Gut histology

Gut samples were obtained from the dissected fish and preserved in a buffered 10% formalin solution. For histological analysis, the method described by Chen et al. [30] was followed. Slides were prepared by staining with hematoxylin and eosin (H&E). The measurement of villus height, villus width, and intestinal epithelial muscle thickness was carried out using Image-Pro Plus 6.3 software (Media Cybernetics, Inc.). The slides were inspected under a Nikon ECLIPSE 80i microscope (Nikon Corporation, Kanagawa, Japan) to capture the necessary images and perform the measurements. This histological analysis allowed the assessment of the structural characteristics of the gut tissue.

### Statistical analysis

The data collected for all parameters are expressed as the mean ± standard error of the mean (SEM). Statistical analysis was conducted using one-way analysis of variance (ANOVA), followed by post hoc comparisons of means using Tukey’s test. The statistical analysis was performed using SPSS software (Version 16; SPSS Inc., Chicago, IL, USA). A significance level of *P<0*.*05* was considered to determine significant differences between the groups.

## Results

### Growth performance

In this experiment, the fish survival rate was 100% across all groups. The results regarding growth performance are stated in Table 2. A significant effect of diet was observed on growth performance in *C*. *catla*. Final weight, weight gain, feed efficiency, and feed conversion ratio were significantly (*P<0*.*05*) reduced when the CSM inclusion level was increased above 50% in the diet. Similarly, the specific growth rate of *C*. *catla* was statistically similar in the 0CSM, 25CSM, and 50CSM dietary groups but significantly (*P<0*.*05*) reduced when 75% or 100% CSM was used as a replacement for SBM. There was no significant effect of replacing SBM with CSM on the viscerosomatic index (VSI), while the hepatosomatic index (HSI) was significantly (*P<0*.*05*) increased. The HSI in the control and 25CSM groups was the same, but it increased with further replacement of SBM with CSM (Table 2).

**Table 2:** Effect of replacement of SBM with CSM on growth performance and feed utilization metrics in *C*. *catla*.

Parameters	Treatments					P-value
	0CSM	25CSM	50CSM	75CSM	100CSM	
Initial weight (g)	41.00 ± 0.58	41.67 ± 0.88	42.33 ± 0.67	40.67 ± 0.67	42.00 ± 0.58	0.441
Final weight (g)	105.33 ± 0.88 ^a^	105.00 ± 1.53 ^a^	101.67 ± 0.33 ^a^	92.00 ± 1.15 ^b^	86.67 ± 1.45 ^b^	0.001
Weight gain (g)	64.33 ± 1.20 ^a^	63.33 ± 0.88 ^a^	59.33 ± 0.33 ^a^	51.33 ± 0.67 ^b^	44.67 ± 2.03 ^c^	0.001
PWG (%)^1^	57.03 ± 4.73 ^a^	52.09 ± 2.89 ^a^	40.25 ± 3.04 ^ab^	26.27 ± 1.90 ^b^	06.52 ± 6.28 ^c^	0.001
Feed intake (g)	82.00 ± 0.58 ^a^	81.33 ± 0.67 ^ab^	79.00 ± 0.58 ^bc^	77.00 ± 0.58 ^c^	74.00 ± 0.58 ^d^	0.001
FI (%)^2^	0.71 ± 0.01 ^a^	0.71 ± 0.02 ^a^	0.74 ± 0.001 ^a^	0.83 ± 0.01 ^b^	0.92 ± 0.04 ^c^	0.001
Feed efficiency	0.78 ± 0.01 ^a^	0.78 ± 0.02 ^a^	0.75 ± 0.01 ^a^	0.67 ± 0.01 ^b^	0.60 ± 0.02 ^c^	0.001
FCR^3^	1.28 ± 0.02 ^a^	1.28 ± 0.03 ^a^	1.33 ± 0.01 ^a^	1.50 ± 0.01 ^b^	1.66 ± 0.06 ^c^	0.001
SGR (%/day)^4^	1.05 ± 0.02 ^a^	1.03 ± 0.01 ^a^	0.97 ± 0.01 ^ab^	0.91 ± 0.01 ^b^	0.80 ± 0.03 ^c^	0.001
VSI^5^	7.80 ± 0.23	8.03 ± 0.20	8.13 ± 0.16	7.89 ± 0.22	7.97 ± 0.17	0.786
HSI^6^	1.38 ± 0.07 ^a^	1.57 ± 0.01 ^ab^	1.69 ± 0.02 ^a^	1.80 ± 0.01 ^a^	1.77 ± 0.10 ^a^	0.002

*0CSM: control without any CSM, 25CSM, 50CSM, 75CSM and 100CSM contain 25, 50, 75, and 100% CSM as replacement of SBM, respectively. Values are means of three replicates and presented as the means ± SEMs. Values in a row with different superscripts (a-c) differ significantly (*P<0*.*05*).

^1^PWG (Percentage Weight Gain)

^2^FI (Feed Intake)

^3^FCR (Feed Conversion Ratio)

^4^SGR (Specific Growth Rate)

^5^VSI, viscerosomatic index

^6^HSI, hepatosomatic indices.

### Body composition

The body composition results are presented in Table 3. The results showed that there was no significant (*P>0*.*05*) effect of substituting SBM with CSM on the body composition (dry matter, crude protein, ether extraction and total ash) of *C*. *catla*.

**Table 3:** Effect of replacement of SBM with CSM on body composition in *C*. *catla*.

Parameters (%)	Treatments					P-value
	0CSM	25CSM	50CSM	75CSM	100CSM	
Dry matter	75.60 ± 0.28	76.13 ± 0.54	75.42 ± 0.90	74.99 ± 0.92	75.41 ± 0.42	0.817
Crude protein	15.54 ± 0.28	15.55 ± 0.31	15.53 ± 0.24	15.54 ± 0.28	15.42 ± 0.21	0.996
Ether extract	25.20 ± 0.17	25.51 ± 0.29	25.35 ± 0.27	25.82 ± 0.19	24.79 ± 0.10	0.065
Total ash	3.08 ± 0.28	3.08 ± 0.33	3.13 ± 0.43	3.22 ± 0.47	3.22 ± 0.32	0.103

*0CSM: control without any CSM, 25CSM, 50CSM, 75CSM and 100CSM contain 25, 50, 75 and 100% CSM as replacement of SBM, respectively. Values are the mean of three replicates and presented as the means ± SEMs.

### Muscle amino acid profile

The findings regarding the amino acid profile of muscle samples in *C*. *catla* are presented in Table 4. The data demonstrate that the levels of most amino acids remained unchanged by the dietary treatments. However, the levels of arginine, phenylalanine, glycine, tyrosine, and total amino acids (TAAs) were significantly (*P<0*.*05*) affected by the replacement of SBM with CSM. The levels of these amino acids decreased as the dietary inclusion levels of CSM increased, except for alanine.

**Table 4:** Effect of replacement of SBM with CSM on the muscle amino acid profile of *C*. *catla*.

Parameters (g/kg)	Treatments					P-value
	0CSM	25CSM	50CSM	75CSM	100CSM	
Methionine	22.6 ± 0.14	22.4 ± 0.07	22.4 ± 0.09	22.4 ± 0.10	22.2 ± 0.17	0.419
Lysine	64.4 ± 0.10	64.3 ± 0.03	64.2 ± 0.04	64.3 ± 0.03	64.2 ± 0.04	0.396
Threonine	30.3 ± 0.12	30.2 ± 0.03	30.3 ± 0.09	30.2 ± 0.03	30.2 ± 0.06	0.388
Arginine	75.7 ± 0.06 ^a^	75.1 ± 0.06 ^b^	75.2 ± 0.04 ^b^	75.3 ± 0.02 ^b^	75.2 ± 0.03 ^b^	0.001
Valine	35.6 ± 0.12	35.3 ± 0.13	35.1 ± 0.03	35.3 ± 0.13	35.2 ± 0.01	0.077
Leucine	59.3 ± 0.09	59.2 ± 0.03	59.3 ± 0.12	59.2 ± 0.03	59.3 ± 0.06	0.439
Isoleucine	33.2 ± 0.04	33.2 ± 0.04	33.4 ± 0.12	33.3 ± 0.09	33.3 ± 0.09	0.216
Histidine	15.3 ± 0.09	15.3 ± 0.03	15.2 ± 0.07	15.2 ± 0.03	15.2 ± 0.02	0.793
Phenylalanine	32.5 ± 0.01 ^a^	32.4 ± 0.10 ^ab^	32.3 ± 0.10 ^ab^	32.2 ± 0.06 ^b^	32.1 ± 0.03 ^b^	0.012
Serine	29.3 ± 0.09	29.2 ± 0.01	29.3 ± 0.10	29.2 ± 0.03	29.4 ± 0.08	0.473
Proline	56.4 ± 0.03	56.3 ± 0.04	56.3 ± 0.03	56.2 ± 0.02	56.3 ± 0.04	0.498
Aspartic acid	80.5 ± 0.08	80.2 ± 0.01	80.3 ± 0.11	80.3 ± 0.09	80.3 ± 0.9	0.150
Alanine	53.2 ± 0.04	53.2 ± 0.01	53.1 ± 0.04	53.3 ± 0.13	53.2 ± 0.01	0.070
Glutamic acid	132.5 ± 0.20	132.2 ± 0.01	132.4 ± 0.14	132.4 ± 0.12	132.2 ± 0.04	0.239
Glycine	64.6 ± 0.03 ^a^	64.3 ± 0.02 ^b^	64.2 ± 0.01 ^b^	64.3 ± 0.03 ^b^	64.1 ± 0.04 ^b^	0.001
Tyrosine	26.5 ± 0.12 ^a^	26.2 ± 0.04 ^b^	26.3 ± 0.03 ^ab^	26.3 ± 0.04 ^ab^	26.1 ± 0.01 ^b^	0.006
TAA	812.1 ± 0.14 ^a^	809.1 ± 0.32 ^b^	809.6 ± 0.24 ^b^	809.8 ± 0.29 ^b^	809.1 ± 0.06 ^b^	0.001

*0CSM: control without any CSM, 25CSM, 50CSM, 75CSM and 100CSM contain 5, 50, 75 and 100% CSM as replacement of SBM, respectively. Values are the mean of three replicates and presented as the means ± SEMs. Values in a row with different superscripts (a-b) differ significantly (*P<0*.*05*).

### Intestinal digestive enzyme activities

Table 5 presents the effects of replacing SBM with CSM on digestive enzyme activity in *C*. *catla*. The results indicate a significant (*P<0*.*05*) effect of the diets on the activities of different digestive enzymes in *C*. *catla*. As the dietary levels of CSM increased, the activities of all digestive enzymes decreased. Amylase activity in the control group (90.87 U/g) was significantly higher than that in all other groups, with the lowest activity observed in the 100CSM group (68.55 U/g). While the activities of protease and lipase in the 25CSM group were statistically similar to those in the control group (0CSM), their activities decreased significantly as the dietary inclusion levels of CSM increased above 25%.

**Table 5:** Effect of replacement of SBM with CSM on digestive enzyme activities in *C*. *catla*.

Parameters	Treatments					P-value
	0CSM	25CSM	50CSM	75CSM	100CSM	
Amylase (U/g)	90.87 ± 0.16 ^a^	83.32 ± 0.24 ^b^	74.64 ± 0.64 ^c^	73.75 ± 0.74 ^c^	68.55 ± 0.30 ^d^	0.001
Protease (U/mg)	55.83 ± 0.18 ^a^	54.93 ± 0.05 ^a^	52.80 ± 0.22 ^b^	48.55 ± 0.30 ^c^	46.76 ± 0.69 ^d^	0.001
Lipase (U/g)	34.18 ± 0.62 ^a^	30.57 ± 0.39 ^ab^	26.83 ± 0.30 ^b^	21.81 ± 1.66 ^c^	20.89 ± 0.40 ^c^	0.001

*0CSM: control without any CSM, 25CSM, 50CSM, 75CSM, and 100CSM contain 25, 50, 75, and 100% CSM as replacement of SBM, respectively. Values are the mean of three replicates and presented as the means ± SEMs. Values in a row with different superscripts (a-c) differ significantly (*P<0*.*05*).

### Blood biochemical indices

ALT and AST levels in the blood biochemical indices were significantly (*P<0*.*05*) enhanced by replacing SBM with CSM, while the values of ALP remained unaffected (P>0.05) (Table 6). The lowest values of all three indices (ALT 250.95 U/L, AST 42.79 U/L, and ALP 74.33 U/L) were observed in the control group (0CSM), while the highest values were observed in the 100CSM group (ALT 409.91 U/L, AST 81.89 U/L, and ALP 75.33 U/L).

**Table 6:** Effect of replacement of SBM with CSM serum biochemical indices in *C*. *catla*.

Parameters (U/L)	Treatments					P-value
	0CSM	25CSM	50CSM	75CSM	100CSM	
ALT^1^	250.95 ± 2.82 ^d^	300.31 ± 0.44 ^c^	328.16 ± 2.25 ^b^	395.08 ± 2.57 ^a^	409.91 ± 2.55 ^a^	0.001
AST^2^	42.79 ± 1.34 ^e^	59.17 ± 0.58 ^d^	64.36 ± 0.62 ^c^	76.43 ± 0.64 ^b^	81.89 ± 1.66 ^a^	0.001
ALP^3^	74.33 ± 0.33	75.33 ± 0.33	75.66 ± 0.67	76.33 ± 0.88	75.33 ± 0.33	0.786

*0CSM: control without any CSM, 25CSM, 50CSM, 75CSM, and 100CSM contain 25, 50, 75, and 100% CSM as replacement of SBM, respectively. Values are the mean of three replicates and presented as the means ± SEMs. Values in a row with different superscripts (a-e) differ significantly (*P<0*.*05*).

^1^ALT: alanine aminotransferase

^2^AST: aspartate aminotransferase

^3^ALP: alkaline phosphatase activity.

### Antioxidant enzyme activity

The results regarding antioxidant enzyme activities in the serum are stated in Table 7. The results showed a significant (*P<0*.*05*) decreasing effect of replacing SBM with CSM on the activities of MDA and CAT, while the activities of SOD remained unaffected (*P>0*.*05*) in *C*. *catla*. The activities of MDA and CAT were statistically similar in the control and 25CSM groups, while they decreased with higher replacement levels of CSM in the diet.

**Table 7:** Effect of replacing SBM with CSM on antioxidant enzyme activities in the serum of *C*. *catla*.

Parameters	Treatments					P-value
	0CSM	25CSM	50CSM	75CSM	100CSM	
MDA (μmol/mg protein)^1^	4.52 ± 0.04 ^a^	4.22 ± 0.02 ^a^	3.67 ± 0.14 ^b^	3.72 ± 0.12 ^b^	3.48 ± 0.13 ^b^	0.001
SOD (U/mg protein)^2^	15.01 ± 0.24	14.63 ± 0.29	14.55 ± 0.21	14.81 ± 0.19	14.35 ± 0.16	0.340
CAT (U/mL)^3^	4.59 ± 0.03 ^a^	4.63 ± 0.06 ^a^	3.80 ± 0.13 ^b^	3.73 ± 0.01 ^b^	3.50 ± 0.18 ^b^	0.001

*0CSM: control without any CSM, 25CSM, 50CSM, 75CSM and 100CSM contain 25, 50, 75 and 100% CSM as replacement of SBM, respectively. Values are the mean of three replicates and presented as the means ± SEMs. Values in a row with different superscripts (a-b) differ significantly (*P<0*.*05*).

^1^MDA: malondialdehyde

^2^SOD: superoxide dismutase

^3^CAT: catalase.

### Intestinal morphology

The results related to intestinal morphology are presented in Table 8. The highest value of the villus height to villus width ratio (VH/VW) was observed in the 25CSM group, followed by the 0CSM and 50CSM groups. However, a further increase in CSM levels resulted in a significant (*P<0*.*05*) reduction in the VH/VW ratio. Similarly, the thickness of the Tunica muscularis (μm) was significantly reduced by using CSM as a replacement for SBM in the diet of *C*. *catla*.

**Table 8:** Effect of replacing SBM with CSM on the gut morphology of *C*. *catla*.

Parameters	Treatments					P-value
	0CSM	25CSM	50CSM	75CSM	100CSM	
VH/VW (μm)^1^	8.9 ± 0.07 ^b^	10.2 ± 0.09 ^a^	8.8 ± 0.04 ^b^	6.5 ± 0.05 ^b^	5.3 ± 0.04 ^b^	0.001
Tunica Muscularis (μm)	106.88±0.97 ^a^	78.45±0.45 ^b^	75.15±0.65 ^b^	54.45± 0.24 ^c^	49.24±0.69 ^c^	0.001

*0CSM: control without any CSM, 25CSM, 50CSM, 75CSM and 100CSM contain 25, 50, 75 and 100% CSM as replacement of SBM, respectively. Values are the mean of three replicates and presented as the means ± SEMs. Values in a row with different superscripts (a-b) differ significantly (*P<0*.*05*). ^1^VH: villus height; ^1^VW: villus width.

## Discussion

The findings of the current investigation support the conclusion that soybean meal (SBM) can be replaced with CSM in the diet of *C*. *catla* up to 50% without significant negative effects on growth-related parameters and other metrics. However, further replacement of SBM with CSM beyond 50% had a negative impact on these parameters. Therefore, it is recommended to use a maximum of 50% CSM as a replacement for SBM in the diet of *C*. *catla* to maintain optimal growth performance and other biological parameters. Therefore, *C*. *catla* could not be reared on a diet containing only CSM as a replacement for SBM, and the results are supported by previous results that stated that SBM could be partially replaced with CSM in the diet of hybrid tilapia [5]. Other studies stated that SBM could be effectively replaced with CSM at levels of 58.8% and 40% in the diets of rainbow trout and crucian carp, respectively [7, 12]. All these studies revealed that CSM could partially replace SBM in different species of fish but could not be included up to 100%. In addition, the dietary replacement level of SBM with CSM also depends upon different factors, including the nutritive value of CSM being used in the experiment [31], which depends on the processing of cotton seed for oil extraction and the species and development stage of fish [32]. Other possible reasons for the reduced growth performance of *C*. *catla* at CSM inclusion levels higher than 25% could be the presence of high free gossypol levels in CSM [33]. As stated by Cheng and Hardy [15], the presence of 165–220 mg/kg free gossypol significantly reduced growth performance in juvenile rainbow trout. Gossypol molecules bind with lysine and make it unavailable to animals, which could be the reason for the stunted growth observed in many previous studies [34]. Protein sources selected on the basis of the presence and bioavailability of essential amino acids [35] and a lower index of essential amino acids (EAAI) could be another reason for lower growth performance at higher dietary CSM levels in carp when requirements are not met.

Additionally, the significant effects of using CSM as a replacement for SBM on the amino acid profile of *C*. *catla* might be due to the difference in the EAAI balance of SBM and CSM or the lower EAAI of CSM and digestibility coefficient variation in protein and EAAs in the respective ingredients, thus failing to provide a ‘true protein’ comparison [36]. In essence, diets should be ideally formulated on a digestible protein and amino acid basis when reliable coefficients of digestibility exist. There was little variation overall in the muscle tissue profile with dietary cottonseed level. This may be somewhat expected given the consistent nature of major muscle proteins such as myosin and actomyosin. A comprehensive review of the muscle nutrient composition of fish was presented by Renata et al. [37]. However, the very small but significant alteration in phenylalanine, glycine, and tyrosine at 100% CSM substitution may be attributable to the metabolic shifts in amino acid synthesis involving mainly nonessential amino acids. Tyrosine can spare the requirement for its synthesis from phenylalanine by hydroxylation, and its interactions in fish were described recently for the stinging catfish (*Heteropneustes fossilis*) by Sayed et al. [38]. Arginine is an essential amino acid for muscle protein development and was not supplemented in the experimental diets for carp but was held constant in muscle tissue at all CSM levels evaluated [39].

The present study observed a significant effect of replacing SBM with CSM on the activities of intestinal digestive enzymes in *C*. *catla*. Similar findings have been reported in other studies investigating the replacement of SBM with alternative plant protein sources. Huang et al. [40] found that the replacement of SBM with rapeseed meal decreased the activities of lipase, trypsin, and amylase in black carp. Hu et al. [41] also reported a reduction in the activities of amylase and lipase when SBM was replaced with CSM in the diet of juvenile black carp. The differences in the activities of intestinal digestive enzymes between SBM and CSM could be attributed to the existence of different antinutritional factors in these protein sources. Factors such as high fibre content or elevated levels of free gossypol in CSM at higher inclusion levels may contribute to the lower activities of digestive enzymes [42]. The differences in the activities of intestinal digestive enzymes between SBM and CSM could be attributed to the presence of different antinutritional factors in these protein sources. Factors such as high fibre content or elevated levels of free gossypol in CSM at higher inclusion levels may impart the lower activities of digestive enzymes [42]. The differences in the activities of intestinal digestive enzymes between SBM and CSM could be attributed to the occurrence of different antinutritional factors in these protein sources, interfering with intestinal enzyme secretion and action. Factors such as high fibre content or elevated levels of free gossypol in CSM at higher inclusion levels may contribute to the lower activities of digestive enzymes [42].

Liver functionality is normally assessed by evaluating the activities of blood biochemical indices, including specific hepatic enzymes, AST, ALT, and ALP [43]. The current study showed that the activity of ALP was not affected by using CSM, while the activities of ALT and AST were significantly enhanced by using higher CSM levels in the diet of *C*. *catla*. These results suggested that higher replacement levels of SBM with CSM could damage the liver, which increases the activities of AST and ALT in the circulating blood serum. Future work could involve histocytochemistry to ascertain a more quantitative scale of hepatic damage and cellular (hepatocyte) changes.

The activities of CAT, SOD, and MDA are indicators of antioxidant status in fish and other animals. The results suggested that the activities of SOD were not affected by dietary replacement of SBM with CSM; however, the activities of other enzymes (MDA and CAT) were decreased by increasing the CSM levels in the diet of *C*. *catla*. Decreasing levels of antioxidant enzymes with increasing levels of dietary CSM showed that CSM decreased the antioxidant potential of fish, which might be due to the production of oxygen metabolites such as peroxides (H_2_O_2_) by gossypol present in CSM [13]. CAT works to convert reactive H_2_O_2_ to H_2_O and works as a main primary defense factor of the antioxidant system. Decreasing values of antioxidant enzymes suggested that CSM lowers the defensive power in fish and was possibly caused by increased inflammatory effects at higher inclusion levels, liberating oxygen-related free radicals. This was also observed by Biwei et al. [44] as described previously for their work on red tailed catfish.

The present study demonstrated that the replacement of SBM with CSM in the diet of *C*. *catla* had a significant impact on intestinal morphology. Higher dietary levels of CSM resulted in a decrease in the villus height to villus width ratio and the thickness of the intestinal tunica muscularis [45]. These findings suggest that the adverse effects on intestinal morphology may be attributed to the higher fibre content of CSM. The negative effects of CSM on intestinal morphology can have implications for nutrient assimilation and subsequently affect the growth performance of *C*. *catla*. The reduced growth performance observed at high dietary CSM levels may be attributed to poor nutrient assimilation resulting from alterations in intestinal morphology. Other studies have also investigated the effects of cottonseed meal on gut morphology in fish. Liu et al. [46] conducted a study on silver sillago (*Sillago sihama*) and replaced fish meal with low-gossypol cottonseed meal. They reported adverse changes in intestinal morphology even with the use of low-gossypol cottonseed meal, indicating the sensitivity of fish to this ingredient at moderate to high inclusion levels. With the knowledge gained from this current investigation, it is possible to develop more economically efficient feed formulations for *C*. *catla* by considering the constraints associated with CSM and determining the optimal inclusion level. Furthermore, a better understanding of the physiological and metabolic parameters affected by CSM allows for more effective decision-making and strategy development in feed formulation for *C*. *catla*.

## Conclusion

In conclusion, *C*. *catla* could perform well by using CSM in the diet up to 50% as a replacement for SBM. However, excessive replacement of SBM with CSM negatively affected growth performance due to reduced antioxidant status and intestinal impairment in *C*. *catla*.
